# Lectures on the Diagnosis and Treatment of Diseases of the Lungs

**Published:** 1840-09-26

**Authors:** W. W. Gerhard


### MEDICAL EXAMINER.

DEVOTED TO MEDICINE, SURGERY, AND THE COLLATERAL SCIENCES.

No. 39.] PHILADELPHIA, SATURDAY, SEPTEMBER 26, 1840. [Vol. III.

### LECTURES ON THE DIAGNOSIS AND TREATMENT OF DISEASES OF THE LUNGS.

BY W. W. GERHARD, M. D.

### LECTURE X.--(Continued.)

#### ASTHMA.

The term asthma is extremely vague, and is still used in a very loose sense. It is commonly applied to any condition of the respiratory system in which there is much oppression, especially if the dyspnœa comes on in paroxysms, and is attended with a wheezing noise during the inspiration or expiration. In many of these cases there is sufficient evidence of organic disease in the lungs or heart to account for the difficulty of breathing; hence the term asthma is thus applied merely to a symptom, and does not designate a specific disease. In other cases there is no evidence of any organic alteration; and the asthma then becomes a peculiar disease, characterized by regular symptoms, but without definite lesions; it is therefore to be classed amongst those diseases to which the common designation, nervous, is applied. The term is a vague one; but if we restrict it to functional disorders which present a sufficient regularity of symptoms to identify them, there is little practical objection to it. In the present state of the science, therefore, we are compelled to admit a nervous asthma, and a periodical dyspnœa without organic lesion.

The diseases of the lungs which are attended with paroxysms of difficulty of breathing, are a variety of bronchitis, emphysema, certain rare cases of miliary tubercles, and the presence of large tumours upon the trachea or the larger bronchial tubes. The variety of bronchitis I have already treated of under its appropriate head; it is one of the most painful and harassing to the patient, but at the same time is the most curable variety of asthmatic diseases, for it often yields to the continued use of ipecacuanha, and other remedies of the kind, with appropriate counter-irritants. The probabilities of cure are of course much enhanced by a voyage to a milder climate. Emphysema may be palliated, if not cured; but miliary tubercles is generally the most intractable, and often the most rapidly fatal variety of phthisis. The tumours which give rise to periodic dyspnœa at first, will cause a permanent difficulty of breathing if they increase much in size; they are various scirrhous growths, but more frequently aneurism of the arch of the aorta in adults, and scrofulous enlargement of the bronchial glands in children. The dyspnœa is at first not permanent in these cases, because the obstruction to the passage of the air is not sufficient to cause great difficulty of the respiration without some congestion of the bronchial mucous membrane; this is more and more apt to recur as the disease continues to advance, and the case may readily be mistaken for one of nervous asthma.

After striking those cases of false asthma from the list, we next come to the diseases of the heart which simulate the same disorder. These are quite numerous; indeed, any serious disorder of the heart, which impedes the circulation, may congest the lungs, and, as a necessary consequence, great dyspnœa will result. The oppression will be very nearly in proportion to the difficulty of the circulation through the heart, and must of course be greatest in those cases in which the valves are most obstructed. These diseases constitute some of the most severe cases of those classed under the general head of asthma.

There remains, then, a nervous asthma, which cannot be classed under these heads. This disease, like most other chronic affections, is in a great degree hereditary, and often passes through several members of a family; all, or a large number of the children of one family, are often subject to attacks of it upon exposure to slight exciting causes. These causes are extremely various; but they are in general such as act particularly upon the nerves of the respiration, and produce a slight oppression, even in individuals who are not at all asthmatic; such as the inhalation of deleterious gases, certain perfumes, a heated, and especially a crowded room, changes of temperature, or changes in the barometical conditions of the air, will all occasionally produce the same results. The effects of atmospheric changes which are not connected with temperature, and can only be recognised by a delicate hygrometer or barometer, are very peculiar. A very little difference in the moisture, or in the altitude of a particular spot above the level of the sea, being often sufficient to bring on, or to remove a severe attack of asthma. The change from a lower and more crowded to a higher and more airy part of the same town, will often produce the same effect. These attacks of nervous asthma are often periodic, or at least especially apt to recur at particular seasons of the year, which are not always the same, although the summer is in general more apt to favour the development of the disease than colder weather. But there is no disorder which is proverbially so peculiar in its turn and mode of attack as asthma,—the most opposite conditions will modify the action of the nerves of respiration. These conditions do not, however, vary much with each individual; they are generally sufficiently regular, but they are extremely different with different persons who seem to offer the same variety of the disorder. This idiosyncracy is not more remarkable than that which is observed in relation to many other functions of the body, especially the digestive, and is of course equally inexplicable.

The symptoms of nervous asthma are similar in this respect, that all who are affected with the disease are liable to sudden and violent paroxysms of dyspnœa, or to slighter derangement of the respiration; at the same time there are no decided signs of bronchial inflammation. If the respiration be examined, the inspiratory sound is feeble, but there is generally no rhonchus; the wheezing which is occasionally heard at a distance from the patient is produced almost exclusively in the larynx. The rhonchi, and other signs of bronchial irritation, are heard if the attack is accidentally complicated with acute bronchitis.

Paroysms of true asthma terminate by a gradual decline, or as in the variety termed asthmatic bronchitis, the attack is not relieved until a free secretion of glairy liquid from the bronchial membrane takes place; in either case the disorder is singularly apt to return in a short time upon a renewal of its exciting causes.

The diagnosis of the disease is, like the prognosis, exceedingly simple. The disorder may always be recognised by the presence of the periodical dyspnœa, and the absence of any decided evidence of structural change. The prognosis is, on the whole, highly favourable; for few cases of the kind terminate unfavourably, but, like the asthma which arises from emphysema, the disease is exceedingly difficult to remove. At the same time the affection is so peculiar in its nature that it often ceases abruptly, without the slightest assignable cause; and at other times, an apparently insignificant impression made upon the nervous system, either directly on the nervous expansions, or indirectly through the medium of the imagination, will often stop a paroxysm, or postpone one for a long period. The prognosis, therefore, is peculiar; and it is very necessary to be guarded in our promises of cure, or in our anticipations of an unfavourable result when the case is most unpromising.

In most patients asthma may be greatly relieved by attending to the exciting causes of the disease, and carefully avoiding them when practicable. This is often less difficult than it would appear to be at first sight; for a very slight change of residence from one situation to another in the same city, or district of country, will often suffice. Sometimes a more decided change becomes necessary, at least at the season of the year when the disorder is most apt to recur, and every patient is not fitted to decide as to the proper change of situation. In the same way a change of occupation, or even the avoidance of certain departments of a particular business, will often succeed. If these changes fail, and the patient is willing to make the sacrifice, a more decided change is advisable; and, in making it, the warm, moist regions of the sea-side, will generally be found preferable to the drier and more hilly country.

The hygienic peculiarities not connected directly with the condition of the air, are less certain in asthma than in most other diseases; and we must here also rely chiefly on the experience of the patient. Those causes which tend to produce bronchitis, favour the development of asthma, although they do not cause it. Hence the avoidance of cold and unnecessary exposure is essential, unless the experience of the patient should teach him that a cold atmosphere agrees better with him than a warmer one. In either case, however, the impression of prolonged cold upon the surface is almost always deleterious, whatever may be its direct influence upon the bronchial mucous membrane. Excesses in diet are also often exciting causes, and the particular perfumes or stimulants of the bronchial membrane which act unfavourably upon the patient, are generally well known to every patient.

There are many modes of arresting the paroxysms, and for the most part the remedies resemble each other only in their general power of producing decided action upon the nerves of respiration. Frequently these remedies are the narcotics; at other times a mere counter-irritant applied between the shoulders will prove effectual in cutting short the paroxysms. In some cases a galvanic plate applied upon the nucha, and communicating with another placed at the point of the sternum, will instantly check an attack of this disorder; and although the cure is not always permanent, yet in some instances the disease does not return. The nauseants and antiphlogistics, which are often useful in emphysema, are sometimes equally effectual in arresting the paroxysms. Amongst them the tincture of lobelia is one of the most certain and convenient, but with some stomachs it is oppressive and irritating.

The various narcotics which are from time to time resorted to, for the relief of asthma, may be administered in the usual way, or be inhaled into the lungs, and thus brought directly in contact with the bronchial membrane. Thus stramonium, tobacco, and other remedies of this class, are often smoked with great benefit; and a method performed lately by M. Raspail, is sometimes of advantage. This consists in inhaling the vapour of camphor; a few pieces of it are placed in a quill, and the patient may breathe through it. The slow volatilization of the camphor brings it directly in contact with the lungs.

These means are, however, all palliative, and there is sometimes no certain relief for the disease. A careful study of the exciting causes, and attention to some very simple hygienic precautions, are the most promising means of treatment.

